# Comparison of the Trabecular Titanium Acetabular Shell with Burch–Schneider Cages in Revision Hip Arthroplasty

**DOI:** 10.3390/jcm14124381

**Published:** 2025-06-19

**Authors:** Pawel Kamiński, Jarosław Ambroży, Rafał Obuchowicz

**Affiliations:** 1 Andrzej Frycz Modrzewski University, 30-705 Krakow, Poland; pawelkam@mp.pl; 2 Malopolan Orthopaedic and Rehabilitation Hospital, Aleja Modrzewiowa 22, 30-224 Krakow, Poland; 3 Department of Diagnostic Imaging, Jagiellonian University Medical College, 30-663 Krakow, Poland

**Keywords:** trabecular titanium, acetabular reinforcement cage, total hip replacements, acetabular defect

## Abstract

**Objective:** In recent years, a significant increase in the incidence of both total hip arthroplasty and acetabular revision surgery has been observed. A substantial proportion of patients requiring these revision procedures present with major bone deficits and extensive osteolysis. In light of these challenges, this study aims to provide a comprehensive comparison between two commonly utilized methods: trabecular titanium shell implants and Burch–Schneider acetabular reinforcement cages. **Methods:** Participants of both sexes were included through a retrospective review of medical records. The sole inclusion criterion was that the patient had undergone revision hip arthroplasty using either Burch–Schneider acetabular reinforcement cages or Regenerex trabecular titanium shell within the past 18 years. No exclusion criteria were applied concerning patient age, laterality, ethnicity, or post-operative status. Each patient was evaluated based on nine predictive factors, including the Paprosky classification, duration of surgery, perioperative blood loss, number of bone grafts and screws used, as well as pre- and post-operative Harris Hip Score (HHS) and Visual Analogue Scale (VAS). **Results:** A total of 220 patients were included in the analysis, with 75% (*n* = 165) comprising the group treated with trabecular titanium implants and 25% (*n* = 55) treated with Burch–Schneider cages. The use of Regenerex trabecular titanium was associated with a 32.40% (*n* = 23.13 mL) reduction in bone graft tissue required and a 13.7% (*n* = 0.59) increase in the number of screws needed. Additionally, the trabecular titanium group experienced a 15.93% (*n* = 179.64 mL) reduction in perioperative blood loss compared to the Burch–Schneider cage group. The other parameters analyzed in the study did not demonstrate statistical significance. **Conclusions:** The use of a trabecular titanium acetabular shell may be an effective option, particularly in patients with severe acetabular deficits, as it provides favorable clinical and radiological outcomes. Additionally, it reduces the number of bone grafts required and allows for faster and more immediate partial weight-bearing on the operated limb.

## 1. Introduction

Total hip arthroplasty (THA) has become increasingly prevalent in recent years, underscoring its status as a widely adopted procedure in orthopedic surgery [1,2,3]. Correspondingly, there has been a significant rise in the incidence of revision surgeries, including acetabular revision surgeries and THA revision procedures [1,2,3]. A major challenge encountered in these cases is the presence of extensive bone loss due to osteolysis, which frequently precludes the stable fixation of the acetabular component. This condition necessitates complex acetabular reconstruction to restore joint stability and function [4,5,6,7,8,9].

The use of autogenous grafts for acetabular reconstruction is promising due to their durability, which allows for quick incorporation. Additionally, the risk of transmitting infectious diseases is eliminated. The main limitation of using this method is the possibility of taking a sufficient number of grafts. It is dictated by the anatomical limitations of the collection site. Another disadvantage is the potential complications in healing an additional wound and the risk of causing chronic pain at the donor site [10,11]. In turn, heterogeneous transplants are available in unlimited quantities. However, they have a reduced osteoconductive and osteoinductive potential, which in turn reduces the chance of complete incorporation and thus increases the risk of implant loosening [12]. The use of allografts also generates a greater risk of surgical site infection compared to the use of autogenous grafts [13].

One potential strategy to address these challenges is the combined use of bone grafts and Burch–Schneider acetabular reinforcement cages in revision arthroplasty. The Burch–Schneider cage offers several advantages, including improved mechanical stability, protection of the bone graft from excessive load, and enhanced conditions for graft incorporation [14,15,16,17]. These features contribute to better restoration of the hip center of rotation and improved joint biomechanics. However, the use of Burch–Schneider cages is associated with certain disadvantages, such as the risk of mechanical failure (e.g., cage loosening or breakage), graft resorption over time, technical difficulty in achieving optimal positioning of the cage, and potential limitations in future revision options [14,15,16,17,18].

The drawback of this technique is the need for the patient to avoid weight-bearing on the operated joint until the bone grafts have integrated and remodeled, a process that typically takes 2–3 months depending on the volume of grafts used [19,20,21].

The advent of trabecular metal, particularly trabecular titanium, has partially addressed these limitations. Titanium has been utilized in medical applications since the 1970s and is well-established for its high biocompatibility [22,23] Trabecular titanium, characterized by its porous structure, promotes angiogenesis and the formation of new bone tissue [24]. In vitro studies have further demonstrated the enhanced osteointegration properties of this material, making it particularly advantageous for orthopedic applications [23,25].

Moreover, in vivo studies in humans have shown rapid integration of both bone tissue and blood vessels within two weeks post-implantation, with significant host tissue overgrowth observed shortly thereafter [25,26]. These properties have contributed to the widespread adoption of trabecular titanium in acetabular components and other elements of acetabular revision surgery elements used in revision arthroplasty, surpassing the application of Burch–Schneider cages [27].

Recovery with Burch–Schneider cages typically involves longer weight-bearing restrictions due to the need for bone graft integration, while trabecular titanium implants often allow for earlier partial weight-bearing.Comparative studies of Burch–Schneider acetabular reinforcement cages (BS-APCs) Zimmer Biomet, Warsaw, IN, USA and Regenerex Smith & Nephew, in Memphis, TN, USA.

Trabecular titanium implants reveal significant differences in clinical outcomes, survival rates, and complication profiles. BS-APCs provide robust mechanical support in severe acetabular bone loss but require extended non-weight-bearing periods and are associated with notable risks, including aseptic loosening, infection, and dislocation, with a 20-year survival rate of 63% (Chougle et al., 2006 [28]). Short-term survival for BS-APCs was 90.6% at 10 years, with a 15.1% failure rate and a 21.2% complication rate reported by [29]. In contrast, Regenerex implants, featuring a highly porous structure that enhances osteointegration and angiogenesis, enable earlier weight-bearing and have demonstrated 100% short-term survival at 2.6 years with no reported aseptic loosening [30]. These implants also showed a 32.4% reduction in bone graft use, a 15.93% decrease in perioperative blood loss, and significantly lower complication (0.8%), infection (0.4%), and dislocation (0.2%) rates [30]. Despite these favorable short-term results, the absence of mid- and long-term data for Regenerex necessitates cautious interpretation when considering their long-term efficacy.

Despite the availability of several studies evaluating Burch–Schneider cages and trabecular titanium implants individually, direct comparative analyses between these two strategies in patients with moderate to severe acetabular defects are scarce. This study aims to fill this gap by providing a real-world comparative evaluation to guide implant selection in complex revision hip arthroplasty.

The objective of this study was to compare the clinical outcomes and perioperative parameters of two acetabular reconstruction strategies in revision hip arthroplasty: the Burch–Schneider reinforcement cage and the Regenerex trabecular titanium shell. Specifically, we aimed to evaluate their effectiveness in managing moderate to severe acetabular defects (Paprosky types 2B–3A) by analyzing implant survival, complication rates, and rehabilitation outcomes.

## 2. Material and Methods

The study included 220 females and males, ranging from 32 to 89 years old, who have undergone revision hip joint arthroplasty (Table 1).

All patients qualified for the study had defects ranging from 2B to 3A according to the Paprosky classification [31].

Before the operation, patients completed a self-assessment questionnaire and provided informed consent. Patients were informed about the procedure, emphasizing its invasive nature.

To ensure the repeatability of the results, an orthopedist with at least six years’ experience in hip replacement procedures was personally involved in the operations and personally responsible for the process.

This protocol was approved by the institutional review board and approved by the Institutional Ethics Committee of the OIL in Cracow nr 103/KBL/OIL/2024, dated 12 December 2024. The procedure and patient management adhered to Good Medical Practice and the Helsinki Declaration

This investigation was retrospective in nature, with patient allocation to implant groups based on clinical indications rather than randomization.

This retrospective study was reported in accordance with the STROBE (Strengthening the Reporting of Observational Studies in Epidemiology) guidelines. A completed STROBE checklist is provided as Supplementary Materials to this article.

**Figure 1:**
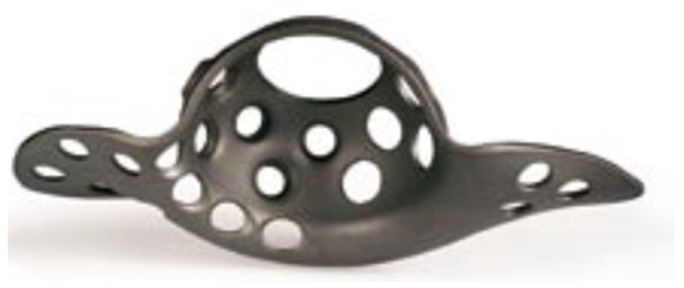
Burch–Schneider acetabular reinforcement cage (Protek, Berne, Switzerland).

The chosen sample was then divided into a first group comprising 55 people of mean age 71.4 years old (SD = 10.5), who underwent operations with the use of Burch–Schneider acetabular reinforcement cages (Protek, Berne, Switzerland) (Figure 1).

**Figure 2:**
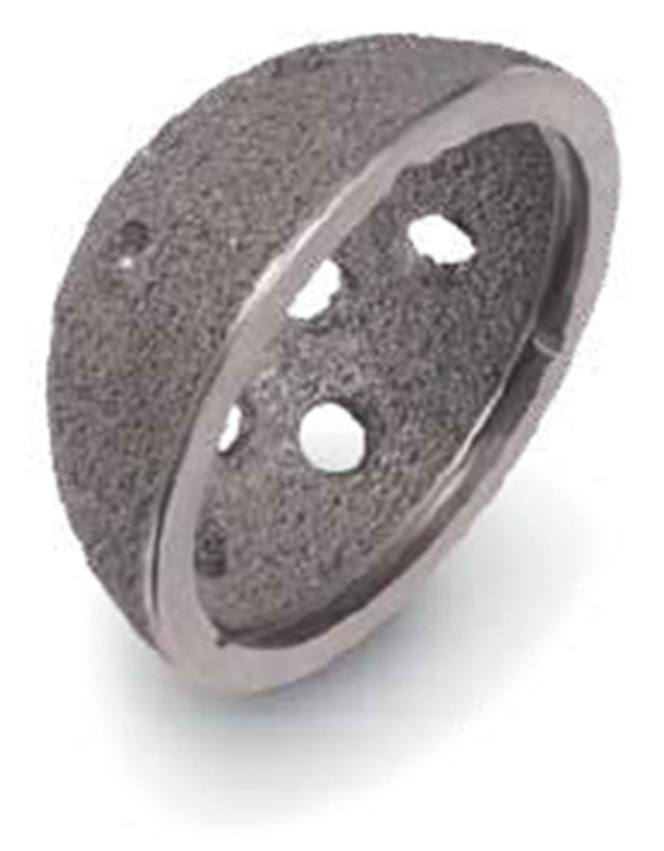
Regenerex trabecular titanium shell (Biomet).

The second group consisted of 165 people of mean age 68.2 years (SD = 12.3), for whom Regenerex trabecular titanium shells (Zimmer Warsaw, IN, USA) were used (Figure 2 and Table 2).

All of the proceeding patients underwent hip joint arthroplasty, during which they were operated on by the anterolateral approach.

The Burch–Schneider acetabular reinforcement cage (Protek, Berne, Switzerland) (Figure 1) consists of a cup with superior and inferior flanges placed accordingly on the iliac and ischium bones. Flanges and cup have holes for additional stabilization by self-tapping screws. Use of this kind of implant demands bone grafting to fill in bone lesions. Rebuilding via grafts supports the final implant.

**Figure 3:**
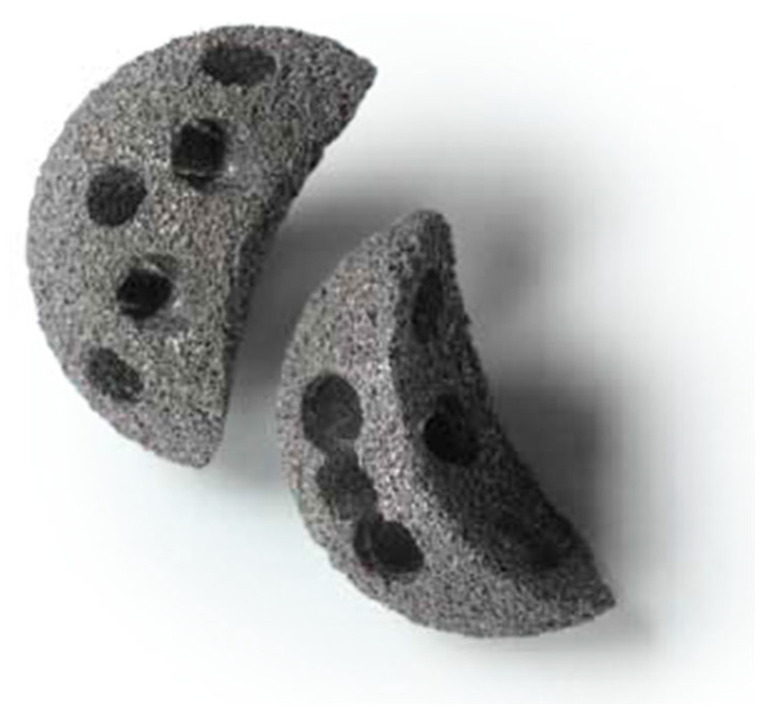
Semilunar titanium implants to fill in defects of the acetabular dome (Biomet).

The Regenerex shell (Zimmer) (Figure 2) is a titanium implant of 67% porosity—which is especially important from the standpoint of the angiogenesis process taking place in bone tissue [22]. The whole system consists of a modular acetabular fragment varying from 48 mm to 72 mm in diameter. It can be implemented with a semi-lunar piece, which enables filling of possible deficits of the acetabulum (Figure 3).

All of the elements contain holes for primary stabilization by self-tapping screws ranging from 15 mm to 50 mm long.

The technique used during our revision hip arthroplasty procedures was identical to the one described by the manufacturer, meaning that the screws were used for ring placement, with initial stabilization of the press-fit implant.

Before surgery, a thorough evaluation was performed, including imaging studies like X-rays, to assess the extent of bone loss with the use of Paprosky classification [31] and the condition of the previous implant. The possible loosening of the implant was classified, either as displacement of the implant or as the lysis area consistent with Charnley and Dellee criteria [32].

Based on these assessments, selection of the appropriate implant, i.e., Burch–Schneider Cage or Regenerex trabecular titanium shell, was made. The final choice was made after intraoperative evaluation of bone lesions.

### 2.1. Procedure Description

The patients were placed under regional anesthesia (such as a spinal block) to ensure they remained pain-free during the procedure. All operations were performed using the anterolateral approach. In each case, material was collected for microbiological testing. The hip endoprosthesis was carefully dislocated to access the acetabular cup. Both the femoral head and the acetabular cup were removed. All old cement, debris, and soft tissues were meticulously debrided. A final verification of acetabular bone defects was then performed.

In cases of significant bone loss, allogenic bone grafts were used to fill the defects. The acetabulum was subsequently prepared to receive the new implant.

For implantation:

The Burch–Schneider Cage was positioned into the acetabulum to reinforce the bone and provide a stable base for the new acetabular cup. Screws were used to secure the cage to the pelvis, ensuring stable fixation.

The Regenerex Trabecular Titanium Shell was placed into the acetabulum. Its porous structure facilitated primary stability. Screws were used as needed to secure the implant.

Once either the reinforcement cage or the titanium shell was securely in place, a new polyethylene acetabular cup for a 28 mm or 32 mm head was cemented in position. After relocation of the new femoral head, joint stability and range of motion were carefully assessed intraoperatively.

After closure of the gluteal muscles, the remaining soft tissue layers were sutured meticulously, and the skin was closed with sutures or staples.

Postoperatively, patients with Regenerex implants were instructed to use two crutches with partial weight-bearing on the operated limb, starting with approximately 30% of body weight, and progressively increasing to full weight-bearing by around three months post-surgery. In contrast, weight-bearing in the Burch–Schneider cage group was delayed for six weeks.

All patients received low molecular weight heparin (LMWH) for up to six weeks postoperatively for thromboprophylaxis.

Radiological evaluation was performed using standard anteroposterior (AP) X-rays taken within the first 24 h postoperatively, and subsequently at 6 weeks, 3 months, and 12 months. For patients with follow-up periods exceeding 12 months, annual radiological assessments were conducted using the same X-ray equipment.

Clinical evaluation was carried out preoperatively and one year postoperatively using the Harris Hip Score (HHS), with scores interpreted as follows: <70 points as poor, 70–79 as fair, 80–89 as good, and 90–99 as excellent. Pain was assessed preoperatively and one year postoperatively using the Visual Analog Scale (VAS).

The mean observation period was 112.5 months, ranging from a minimum of 15 months to a maximum of 209 months.

### 2.2. Perioperative Blood Loss and Transfusion Assessment

Intraoperative blood loss was measured as the total amount of fluid collected in the suction container during surgery, from which the volume of lavage fluid used was subtracted. To account for blood absorbed by surgical drapes and setons, a standard deduction of 200 mL was applied. We did not use the Groos or Mercuriali formulas due to the difficulties associated with precisely estimating the patient’s blood volume, which is necessary to perform the calculations [33]. Postoperative blood loss was assessed based on the total drainage volume collected in the suction bottle from the surgical drain during the first 24 h postoperatively.

The indication for blood transfusion was based on the hemoglobin concentration measured in the morning of the day after surgery (17–22 h). Transfusion of packed red blood cells (PRBC) was performed in patients with hemoglobin levels < 8 g/dL or in cases of clinical symptoms of anemia (e.g., hypotension, tachycardia, hypoxia) at the discretion of the attending physician. Fresh frozen plasma (FFP) was administered in cases of coagulation abnormalities identified by laboratory tests or significant blood loss during surgery.

All transfusion volumes, blood loss amounts, and postoperative drainage volumes were recorded prospectively in the patients’ medical charts and analyzed retrospectively for the purpose of this study.

The results were analyzed statistically with the use of GraphPad Prism 6. Statistical significance was established as α = 0.05. Moreover, the descriptive statistics of qualitative variables consists of absolute and relative frequencies featured in percentage-wise fashion. Quantitative changes were described with the use of mean value, as well as standard deviation (SD), median (Me) and interquartile range (IQR). The Shapiro–Wilk test was used to test the normality of the distribution, with marking of the skewness variables. The pre- and post-operative differences of quantitative variables were compared with Student’s T test for dependent samples. Differences in distribution of quantitative variables between the groups were compared by Student’s T test for independent samples when the distribution of variables was uniform, or with normal distribution or U Mann–Whitney tests in cases where distributions weren’t consistent.

## 3. Results

Acetabular bone loss was analyzed according to Paprosky classification. Among the 55 patients treated with Burch–Schneider cages, 17 had II B defects, 13 had II C defects, 7 had III A defects, 9 had III B defects, and 3 had III C defects. In the Regenerex group, 51 had II B defects, 32 had II C, 41 had III A, 15 had III B, 1 had III C, 23 had II A, and 1 had II B/C (Table 3).

### 3.1. Data of Surgery

#### 3.1.1. A. Duration of Surgery

The mean duration of surgery was 138.18 ± 41.01 min in the Burch–Schneider group and 139.52 ± 42.62 min in the Regenerex group (*p* = 0.84), showing no statistically significant difference between the groups (Table 4).

The comparison between Burch–Schneider Cages and Regenerex implants regarding the duration of surgery revealed no statistically significant difference (*p* = 0.84). Both groups had similar surgery times, indicating that the choice of implant did not substantially affect the duration of the surgery. The intraoperative blood loss between the two groups showed no significant difference (*p* = 0.59), with both implants resulting in comparable amounts of blood loss during surgery.

#### 3.1.2. Intraoperative and Postoperative Blood Loss

The mean intraoperative blood loss was 711.82 ± 422.84 mL for the Burch–Schneider group and 742.73 ± 348.61 mL for the Regenerex group (*p* = 0.59), with no statistically significant difference (Table 4).

### 3.2. Data of Postoperative Follow-Up

#### 3.2.1. Postoperative Blood Loss

Postoperative blood loss was significantly lower in the Regenerex group compared to the Burch–Schneider group (948.12 ± 507.84 mL vs. 1127.64 ± 601.54 mL, *p* = 0.03) (Table 4).

Retransfusion Volumes

There was no significant difference in retransfusion volumes between the two groups. The mean retransfusion volume was 122.55 ± 319.07 mL in the Burch–Schneider group and 81.52 ± 187.18 mL in the Regenerex group (*p* = 0.67) (Table 4).

The analysis of re-transfusion volumes showed no statistically significant difference between the two groups (*p* = 0.67). Both Burch–Schneider Cages and Regenerex implants required similar volumes of blood re-transfusion.

#### 3.2.2. Packed Red Blood Cell (PRBC) and Fresh Frozen Plasma (FFP) Transfusion

The median number of PRBC units transfused was 4 units (IQR 3) for both groups (*p* = 0.57). The median number of FFP units transfused was 1 unit (IQR 2) in the Burch–Schneider group and 2 units (IQR 2) in the Regenerex group, with no significant difference (*p* = 0.11) (Table 4).

Transfusion of Packed Red Blood Cells (PRBC) and Fresh Frozen Plasma (FFP)

There was no significant difference in the median number of PRBC units transfused between the two groups (*p* = 0.57).

The number of FFP units transfused also did not show a statistically significant difference (*p* = 0.11), although there was a trend towards more FFP use in the Regenerex group. This difference was not significant, indicating that the need for FFP transfusion was similar for both implants.

#### 3.2.3. Bone Grafts and Screws

The number of bone grafts used was significantly higher in the Burch–Schneider group (71.38 ± 46.22) compared to the Regenerex group (48.25 ± 40.30) (*p* < 0.01). Conversely, the Regenerex group required significantly more screws (4.48 ± 1.19 vs. 3.89 ± 1.57, *p* < 0.01) (Table 4).

The number of bone grafts used was significantly lower in the Regenerex group compared to the Burch–Schneider cages group (*p* < 0.01).

Conversely, the Regenerex group required a significantly higher number of screws compared to the Burch–Schneider cages group (*p* < 0.01).

Mean number of bone grafts and screws used in patients receiving Burch–Schneider cages versus Regenerex implants: The mean number of bone grafts used was higher in the Burch–Schneider cages group (71.38 ± 46.22) compared to the Regenerex group (48.25 ± 40.30). Conversely, the mean number of screws used was slightly lower in the Burch–Schneider cages group (3.89 ± 1.57) than in the Regenerex group (4.48 ± 1.19). 

#### 3.2.4. Hospitalization Time

There was no significant difference in hospitalization time between groups: the median was 16 days (IQR 8) in the Burch–Schneider group and 15 days (IQR 8) in the Regenerex group (*p* = 0.49) (Table 4).

### 3.3. Data of Last Follow-Up

#### 3.3.1. Preoperative Harris Hip Score (HHS) and Visual Analogue Scale (VAS)

The preoperative mean HHS was significantly lower in the Burch–Schneider group (15.43 ± 9.77) compared to the Regenerex group (25.48 ± 11.75) (*p* < 0.01). The preoperative mean VAS pain score was significantly higher in the Burch–Schneider group (9.02 ± 0.60) than in the Regenerex group (7.22 ± 1.95) (*p* < 0.01) (Table 5).

There was a significant difference in preoperative HHS, with the Regenerex group having a higher mean score (*p* < 0.01). The Harris Hip Scores (HHS) are indicative of the patients’ poor status (HHS < 70) (Table 5).

The Regenerex group also had significantly lower preoperative VAS pain scores (*p* < 0.01).

#### 3.3.2. One-Year Postoperative Harris Hip Score (HHS) and Visual Analogue Scale (VAS)

At one year postoperatively, there was no significant difference between the groups.

The mean HHS was 67.48 ± 33.04 in the Burch–Schneider group and 68.15 ± 23.28 in the Regenerex group (*p* = 0.89).

Similarly, the mean VAS pain scores were comparable: 3.50 ± 2.82 in the Burch–Schneider group versus 3.19 ± 1.89 in the Regenerex group (*p* = 0.8) (Table 6).

One year after surgery, there was no significant difference in HHS between the two groups (*p* = 0.89). Both implants led to similar improvements in hip function, indicating that they are equally effective in this regard.

**Figure 4:**
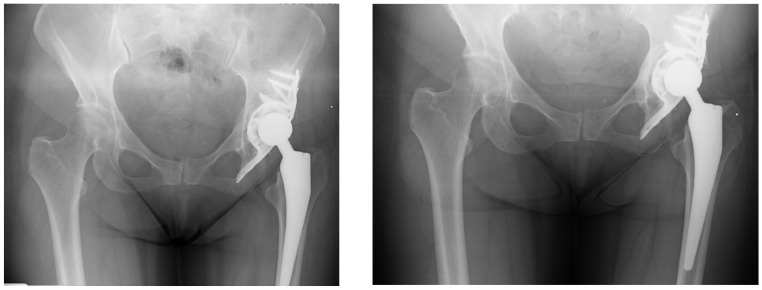
The images (from 2013—**left** and from 2015—**right**) demonstrate the typical phenomenon of bone loss in the least loaded area beneath the dome of the acetabular cage, which is a recognized biological response in such reconstructions.

Similarly, there was no significant difference in postoperative VAS scores (*p* = 0.8), with both groups reporting comparable pain levels one year after surgery (Table 5 and Figure 4).

## 4. Discussion

In this retrospective comparative study involving 220 patients undergoing revision total hip arthroplasty for Paprosky type 2B to 3A acetabular defects, we identified several key distinctions in clinical and perioperative outcomes between the Burch–Schneider acetabular reinforcement cage and the Regenerex trabecular titanium shell. The mean duration of surgery and intraoperative blood loss were statistically comparable between the two implant groups, suggesting a similar level of surgical complexity. However, postoperative blood loss was significantly lower in the Regenerex cohort, a finding potentially attributable to the superior hemostatic properties of trabecular titanium [34]. Transfusion requirements, including packed red blood cells and fresh frozen plasma, did not differ significantly between the groups. Notably, acetabular reconstruction using the Regenerex implant necessitated a significantly smaller volume of bone grafts, albeit requiring a higher number of fixation screws to achieve satisfactory primary stability. Both groups demonstrated clinical improvement postoperatively; however, patients in the Regenerex group reported lower preoperative VAS pain scores and achieved comparable one-year Harris Hip Scores (HHS), but it should be pointed out that the Regenerex group started from a better baseline functional status.

Our results are consistent with the previously published literature. The reduced dependence on bone grafts in the Regenerex group aligns with existing evidence indicating that trabecular titanium implants can replace bone grafts to some extent [35,36]. The enhanced biological integration offered by trabecular titanium, characterized by its high porosity, biocompatibility, and promotion of angiogenesis, contributes to faster osseointegration and facilitates early partial weight-bearing [25,37,38,39,40,41,42]. These properties are further supported by favorable biomechanical characteristics, including physiological elasticity and long-term material durability in revision settings [43,44,45,46]. Additionally, the surface roughness of Regenerex implants augments primary mechanical fixation, enabling a progressive rehabilitation protocol from partial to full weight-bearing [47,48,49,50,51,52,53]. Conversely, rehabilitation following implantation of Burch–Schneider cages is inherently delayed due to the need for graft incorporation prior to load-bearing.

With regard to functional outcomes, our study corroborates prior observations indicating superior early pain relief in patients treated with trabecular titanium implants. The observed improvement in VAS pain scores among Regenerex recipients is consistent with the literature documenting improved patient-reported outcomes following biologically integrated implant use. However, the overall postoperative HHS in both groups was moderate and somewhat lower than values reported in cohorts with better baseline function. For example, Perticarini et al. [52] noted greater improvements in patients with less severe preoperative impairment, underscoring the impact of baseline status on postoperative functional recovery.

Implant survivorship in the Burch–Schneider group was comparable to previously published data. Our observed short-term (90.9%), mid-term (89.1%), and long-term (61.8%) survival rates closely mirror the findings of Malahias et al. [54] (90.6%, 85.6%, and 62%, respectively). The cumulative survival at 7.5 years (69.1%) in our cohort was somewhat lower than the 88.1% reported by Lopez-Torres et al. [55] likely reflecting the more advanced disease stage and higher complexity in our patient population. While aseptic loosening rates were comparable (5.5% vs. 5.6%), our cohort demonstrated higher incidences of periprosthetic joint infection (7.3% vs. 3.8%) and dislocation (12.7% vs. 2.7%), possibly due to poorer baseline joint integrity. In contrast, the Regenerex group showed excellent short-term outcomes, with no cases of aseptic loosening, and markedly lower rates of infection (0.4%) and dislocation (0.2%). Additionally, there was a 32.4% reduction in bone graft usage and a 15.93% reduction in perioperative blood loss relative to historical controls [54,55,56,57,58].

An additional point of interest pertains to ongoing innovations in Burch–Schneider cage design. Recent adaptations, such as the incorporation of trabecular titanium surfaces and the modified positioning of the inferior flange within the ischium, aim to combine the robust mechanical characteristics of traditional cages with the biological advantages seen in modern porous implants [29,45,49,50,51,52,53,54,55,56,57,58,59,60,61]. These hybrid approaches may represent a promising direction in the management of complex acetabular reconstruction cases.

Our study has several strengths. It includes a relatively large sample size and a mean follow-up duration exceeding nine years (112.5 months), enhancing the reliability of long-term outcome assessments. All surgical procedures were performed by experienced orthopedic surgeons using standardized operative techniques, thereby minimizing procedural variability. The inclusion criteria, limited to Paprosky type 2B to 3A defects, ensured a homogeneous patient population representative of challenging but reconstructable acetabular deficiencies. Furthermore, the study adhered to STROBE guidelines and utilized uniform methods for data collection regarding blood loss, transfusion, and rehabilitation metrics.

Certain limitations must be acknowledged. First, the retrospective nature of the study introduces inherent risks of selection bias and limits the ability to control for all confounding variables. Implant allocation was determined intraoperatively based on defect morphology and surgical judgment, precluding randomization. Second, although the follow-up duration was adequate for evaluating the performance of Burch–Schneider cages, it remains insufficient to definitively assess the long-term durability of Regenerex implants. Additionally, preoperative functional status was significantly worse in the Burch–Schneider group, which may have confounded postoperative comparisons. Finally, the absence of advanced imaging modalities, such as CT or radiostereometric analysis, restricts the precision of assessments related to implant osseointegration and migration.

## 5. Conclusions

This study demonstrates that both Burch–Schneider acetabular reinforcement cages and Regenerex trabecular titanium shells are effective options for managing moderate to severe acetabular defects (Paprosky types 2B to 3A) in revision hip arthroplasty. Regenerex implants, characterized by their porous trabecular structure, were associated with reduced postoperative blood loss, decreased bone graft requirements, and earlier weight-bearing, which may facilitate faster rehabilitation without compromising long-term outcomes. Despite higher complication rates, Burch–Schneider cages remain an essential solution for cases involving extensive bone loss or pelvic discontinuity where immediate mechanical reinforcement is critical. Implant selection should therefore be individualized, taking into account defect severity, patient condition, and rehabilitation potential. Our findings suggest that Regenerex implants may offer biological and perioperative advantages in patients eligible for earlier mobilization, although further prospective studies are necessary to confirm their long-term durability compared to traditional reinforcement cages.

Both Regenerex trabecular titanium shells and Burch–Schneider cages have their place in contemporary revision hip arthroplasty. While Regenerex appears to offer biological and perioperative advantages, the Burch–Schneider cage remains indispensable in scenarios of severe bone loss. A personalized approach, informed by defect severity, surgical goals, and patient-specific factors, is essential to achieve optimal clinical and functional results in this challenging patient population.

## Figures and Tables

**Table 1:** Inclusion and exclusion criteria for the proposed procedures.

Inclusion Criteria	Exclusion Criteria
Age ≥ 18 years	Active infection in the hip joint (clinical or microbiological)
Underwent revision total hip arthroplasty (THA)	Ongoing systemic infection or sepsis
Diagnosis of aseptic loosening of the acetabular component	Severe medical comorbidities precluding surgery (e.g., ASA IV–V)
Paprosky classification type 2B to 3A	Paprosky type 1, 2A, or 3C acetabular defects
Underwent surgery using either Burch–Schneider cages or Regenerex trabecular titanium	History of periprosthetic joint infection in the past 6 months
Written informed consent obtained	Incomplete radiological or clinical documentation pre- or postoperatively
Available for a minimum of 15 months of follow-up	Prior radiation therapy to the pelvis
Able to undergo clinical and radiological follow-up (HHS, VAS, X-rays)	Neuromuscular or neurodegenerative disorders affecting lower limbs (e.g., ALS)
Bone grafting required during revision	Severe psychiatric illness impacting compliance
No contraindications for regional or general anesthesia	Pregnancy
	Patients who died within 12 months after the revision procedure
	Patients who were lost to follow-up

**Table 2:** Characteristics of selected groups.

Category	Burch–Schneider Cage Group	Regenerex Trabecular Titanium Group
Series	55 patients	165 patients
Mean Age (years)	71.4 ± 10.5	68.2 ± 12.3
Gender (F/M)	50/5	113/52
Paprosky Defect Types	II A: 10.91% II B: 30.91% II C: 23.64% III A: 12.73% III B: 16.36% III C: 5.45%	II A: 13.93% II B: 30.91% II C: 19.39% III A: 24.85% III B: 9.09% III C: 0.61%
Surgical Procedure	Anterolateral approach; use of Burch–Schneider reinforcement cages with allogenic bone grafts; cemented polyethylene cups	Anterolateral approach; Regenerex titanium shell with modular inserts; minimal bone grafting; cemented polyethylene cups
Mean Duration of Surgery (minutes)	138.18 ± 41.01	139.52 ± 42.62
Mean Intraoperative Blood Loss (mL)	711.82 ± 422.84	742.73 ± 348.61
Post-Operative Follow-Up	Partial weight-bearing delayed for 6 weeks	Early partial weight-bearing from 30% body weight
Mean Hospitalization Time (days)	16 (IQR 8)	15 (IQR 8)
Mean Follow-Up (months)	112.5 months (range 15–209)	112.5 months (range 15–209)
Statistics	Statistical analysis performed using Shapiro–Wilk test for normality, Student’s *t*-test and Mann–Whitney U test; significance set at α = 0.05	Statistical analysis performed using Shapiro–Wilk test for normality, Student’s *t*-test and Mann–Whitney U test; significance set at α = 0.05

**Table 3:** Paprosky classification in distinctive study groups.

Paprosky Classification	Burch–Schneider Cage		Regenerex		*p*
	*n*	%	*n*	%	
**I**	-	-	1	0.61	(*p* < 0.05)
**II A**	6	10.91	23	13.93	
**II B**	17	30.91	51	30.91	
**II C**	13	23.64	32	19.39	
**II B/C**	-	-	1	0.61	
**III A**	7	12.73	41	24.85	
**III B**	9	16.36	15	9.09	
**III C**	3	5.45	1	0.61	

*n*—number of participants, *p* < 0.05 statistically significant value.

**Table 4:** Comparison of perioperative and clinical parameters between Burch–Schneider cage and Regenerex implant groups.

Parameter	*n* (BS Cage)	Mean/Median (BS Cage)	SD/IQR (BS Cage)	*n* (Regenerex)	Mean/Median (Regenerex)	SD/IQR (Regenerex)	*p*-Value
Duration of surgery [min]	55	138.18	41.01	165	139.52	42.62	0.84
Intraoperative blood loss [mL]	55	711.82	422.84	165	742.73	348.61	0.59
Postoperative blood loss [mL]	55	1127.64	601.54	165	948.12	507.84	0.03
Re-transfusion [mL]	55	122.55	319.07	165	81.52	187.18	0.67
PRBC [units]	55	4	3	165	4	3	0.57
FFP [units]	55	1	2	165	2	2	0.11
Number of bone grafts used	55	71.38	46.22	165	48.25	40.3	<0.01
Number of screws used	55	3.89	1.57	165	4.48	1.19	<0.01
Hospitalization time [days]	55	16	8	165	15	8	0.49

**Table 5:** Preoperative Harris Hip Score (HHS) and Visual Analogue Scale (VAS) in patients receiving Burch–Schneider cages versus Regenerex implants. The Burch–Schneider cages group demonstrated a lower mean preoperative HHS (15.43 ± 9.77) compared to the Regenerex group (25.48 ± 11.75), indicating more compromised hip function. Conversely, the mean preoperative VAS pain score was higher in the Burch–Schneider group (9.02 ± 0.60) compared to the Regenerex group (7.22 ± 1.95). These results suggest that patients receiving Burch–Schneider cages presented with worse preoperative clinical status. *p* < 0.05 statistically significant value; *n*—number of participants; Me- median; IQR—interquartile range.

	Burch–Schneider Cage			Regenerex			*p*
	*n*	Mean	SD	*n*	Mean	SD	
**HHS**	43	15.43	9.77	127	25.48	11.75	**<0.01**
**VAS**	42	9.02	0.6	126	7.22	1.95	**<0.01**

**Table 6:** Harris Hip Score (HHS) and Visual Analogue Scale (VAS) one year after surgery in patients treated with Burch–Schneider cages versus Regenerex implants. At one-year follow-up, the mean HHS was 67.48 ± 33.04 in the Burch–Schneider cages group and 68.15 ± 33.28 in the Regenerex group, indicating comparable functional outcomes. The mean VAS pain score was 3.50 ± 2.82 for the Burch–Schneider group and 3.19 ± 1.89 for the Regenerex group. These results suggest similar improvements in hip function and pain reduction across both implant types. *p* < 0.05 statistically significant value; *n*—number of participants; SD—standard deviation.

	Burch–Schneider Rings			Regenerex			*p*
	*n*	Mean	SD	*n*	Mean	SD	
**HHS**	43	67.48	33.04	127	68.15	23.28	0.89
**VAS**	42	3.5	2.82	126	3.19	1.89	0.8

## Data Availability

The dataset generated and analyzed during the current study is not publicly available due to institutional and ethical restrictions but is available from the corresponding author upon reasonable request. Requests for access to the data should be directed to r.obuchowicz@gmail.com. All data will be shared in accordance with applicable data protection and privacy regulations.

## Supplementary Materials

The following supporting information can be downloaded at: https://www.mdpi.com/article/10.3390/jcm14124381/s1, Table S1: STROBE Checklist for Observational Studies.
