# Supplementary material for: Comparison of the Trabecular Titanium Acetabular Shell with Burch–Schneider Cages in Revision Hip Arthroplasty
Source: J Clin Med. 2025 Jun 19;14(12):4381. doi: 10.3390/jcm14124381 (PMC12194563; doi:10.3390/jcm14124381)
Supplement: Supplementary file 1 [file jcm-14-04381-s001.zip › jcm-3599972-supplementary.pdf]

Supplemenary Materials

Table S1: STROBE Checklist for Observational Studies

Section/Topic	Item No.	Recommendation	Manuscript Location
**Title and Abstract**	1	Indicate the study's design with a commonly used term in the title or abstract	Title and Abstract: "retrospective comparative study" added
**Introduction**	2	Explain the scientific background and rationale for the investigation being reported	Introduction section
	3	State specific objectives, including any prespecified hypotheses	End of Introduction, Objectives
**Methods**	4	Present key elements of study design early in the paper	Material and Methods section, paragraph 1
	5	Describe the setting, locations, and relevant dates, including periods of recruitment, exposure, follow-up, and data collection	Material and Methods section
	6	Give the eligibility criteria, and the sources and methods of selection of participants	Material and Methods, Inclusion and Exclusion Criteria
	7	Clearly define all outcomes, exposures, predictors, potential confounders, and effect modifiers. Give diagnostic criteria if applicable	Material and Methods (definitions of outcomes, HHS, VAS)
	8	For each variable of interest, give sources of data and details of methods of assessment (measurement)	Material and Methods: Clinical and Radiological Assessments
	9	Describe efforts to address potential sources of bias	Retrospective design and standardization of surgical procedures by experienced surgeons described
	10	Explain how the study size was arrived at	All eligible patients during the study period were included (retrospective cohort)
	11	Explain how quantitative variables were handled in the analyses; describe which groupings were chosen and why	Material and Methods: Statistical Analysis section
	12	Describe all statistical methods, including those used to control for confounding	Material and Methods: Statistical Analysis section (Student t-test, Mann-Whitney test)
**Results**	13	Report numbers of individuals at each stage of study—e.g., numbers potentially eligible, examined for eligibility, confirmed eligible, included in the study, completing follow-up, and analyzed	Results section, patient numbers provided
	14	Give characteristics of study participants (e.g., demographic, clinical, social) and information on exposures and potential confounders	Results section, Tables 2 and 3

Section/Topic	Item No.	Recommendation	Manuscript Location
	15	Report numbers of outcome events or summary measures	Results, perioperative and clinical outcomes
	16	Report unadjusted estimates and, if applicable, confounder-adjusted estimates and their precision (e.g., 95% confidence intervals). Make clear which confounders were adjusted for and why they were included	Not applicable (pure comparative analysis, no multivariate adjustment performed)
**Discussion**	17	Summarize key results with reference to study objectives	Discussion section
	18	Discuss limitations of the study, taking into account sources of potential bias or imprecision	Discussion, Limitations implicitly discussed (e.g., retrospective design, absence of long-term Regenerex outcomes)
	19	Give a cautious overall interpretation of results considering objectives, limitations, multiplicity of analyses, results from similar studies, and other relevant evidence	Discussion and Conclusion sections
	20	Discuss the generalizability (external validity) of the study results	Discussion, Conclusion sections
**Other Information**	21	State the source of funding and the role of the funders for the present study	Funding section ("no external funding")
